# High‐level seroprevalence against *Leptospira interrogans* serovars among wild foxes, jackals and stray dogs in the North Khorasan Province, Iran

**DOI:** 10.1002/vms3.890

**Published:** 2022-07-15

**Authors:** Kourosh Arzamani, Gholamreza Abdollahpour, Amir Azimian, Alex van Belkum, Hamed Ghasemzadeh‐Moghaddam

**Affiliations:** ^1^ Vector‐Borne Disease Research Center North Khorasan University of Medical Sciences Bojnurd Iran; ^2^ Leptospira Research Laboratory Faculty of Veterinary Medicine Department of Internal Medicine University of Tehran Tehran Iran; ^3^ Department of Pathobiology and Laboratory Sciences North Khorasan University of Medical Sciences Bojnurd Iran; ^4^ Open Innovation & Partnerships BaseClear Leiden The Netherlands

**Keywords:** fox, Iran, jackal, leptospirosis, stray dogs

## Abstract

**Background:**

Leptospirosis is an important, neglected zoonotic disease that affects people and animals in humid (sub)tropical regions. Wild canines carry the pathogen and may contaminate natural resources which may then act as a source of human infection.

**Objectives:**

The study was designed to understand the seroprevalence of leptospirosis among domestic and wild canines in Bojnurd County, Northeast Iran.

**Methods:**

A total of 77 serum samples, comprising 29 sera from asymptomatic wild canines [foxes (*n* = 25) and jackals (*n* = 4)] and 48 sera from asymptomatic stray dogs, was investigated. Serovars were identified and antibody titres were measured by standard microscopic agglutination test (MAT) using serial serum dilutions.

**Results:**

Among all serum samples, 44.1% reacted positively to a *Leptospira interrogans* serovars. The average percentage of positive reactions was higher in stray dogs than in wild canines although this did not reach statistical significance (55.2% and 37.5%, *p = *0.159). Positive reactions with *L. i. Pomona*, *L. i. Australis* and *L. i. Tarasovi* was detected only among jackals and foxes. Among the stray dogs, the highest number of positive sera were for *L. i. Grippotyphosa* (61.1%) and *L. i. Canicola* (50%). The highest titre detected was for *L. i. canicola* (1:1600) in two stray dogs and against *L. i. Icterohaemorrhagiae* and *L. i. Pomona* (1:800) in a single jackal.

**Conclusions:**

The study revealed that leptospirosis is endemic among various canine species in the North Khorasan Province of Iran. Detailed monitoring of canines is necessary for better understanding the epidemiology of infection in our and other Iranian regions.

## Graphical abstract

In the current study, the average percentage of positive reactions among stray dogs (55.2%) was higher than wild canines (37.2%, *P* = 0.159). The study revealed leptospirosis is endemic among various canine species in the North Khorasan Province of Iran. Detailed monitoring of canines suggested.

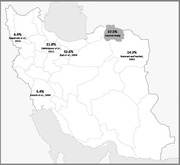

## 1 INTRODUCTION

Leptospirosis, a globally neglected zoonotic disease causing fever for days to weeks, affects people and animals in humid (sub‐)tropical regions (Ullmann & Langoni, 2011). Leptospirosis is mild in 90% of all cases but may generate severe complications in other patients. Although the main symptoms are fever, muscle pain and headaches, the disease can also lead to serious organ failure (kidneys, liver) and haemorrhaging. The disease is spread by a large variety of both wild and domestic animals which are natural reservoirs of *Leptospira* spp. (Adler & de la Peña Moctezuma, 2010). A wide variety of animals host *Leptospira interrogans* and many of these are asymptomatic Leptospira renal carriers. They contaminate the environment by shedding bacteria in their urine and they may develop symptoms only after long incubation periods (Adler & de la Peña Moctezuma, 2010). The precise epidemiology of leptospirosis in a specific niche is defined by the close contact between the particular Leptospira serovars and their specific maintenance hosts (Fratini et al., 2020). The seroprevalence of leptospirosis in humans and different animals in Iran has been studied before (Khalili et al., 2020). Leptospirosis is endemic in the North Khorasan province, as recent studies using the microscopic agglutination test (MAT) unveiled past and present infections among both rodents (Arzamani et al., 2018) and humans (Hashemi et al., 2021). Leptospiral infection, its associated prevalence and the dominant serovars were reported as being different in canines around the world (Ab Rahman et al., 2018; Ambily et al., 2013; Aslantaş et al., 2005; Azocar‐Aedo et al., 2017; Lelu et al., 2015; Samir et al., 2015; Shi et al., 2012). Similar reports on seroprevalence have emerged from different geographic regions of Iran as well (Avizeh et al., 2009; Fahimipour et al., 2021; Jamshidi et al., 2008; Rad et al., 2004; Torkan & Momtaz, 2019). Wild and feral canines carry the pathogen and contaminate the environment including soils, surface waters, streams and rivers where the bacteria can survive for weeks to months. This acts as one of the most significant sources of infection. In the current study *Leptospira* spp. seroprevalence among foxes, jackals and stray dogs in Bojnurd County, Northeast of Iran, was investigated for the first time.

## 2 METHODS AND MATERIALS

### 2.1 Study population and blood sample collection

**FIGURE 1:**
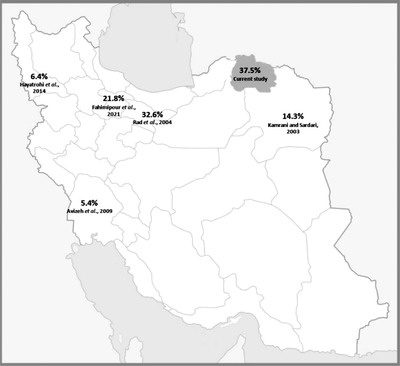
Study location and illustration of the rate of leptospiral positive reactions among canines in different geographic regions of Iran

This study was conducted in the North Khorasan province in the Northeast of Iran (37.47 N and 57.33 E) (Figure 1). In a six months period, from April to September in 2020, stray dogs and wild canines were collected by the municipal animal control department and examined by veterinarians. All asymptomatic animals subjected to this study. A total of 77 blood samples were randomly selected from 29 asymptomatic wild canines [foxes (*n* = 25) and jackals (*n* = 4)] and 48 also randomly selected asymptomatic stray dogs were included in the current study. Five millilitres of blood were collected aseptically from each animal and centrifuged at 3000 rpm for 10 min. Sera were kept at –20°C in micro‐tubes. The sera were transferred to the Leptospira Research Laboratory of the Veterinary Research and Teaching Hospital at the University of Tehran for further analysis while maintaining cold chain management.

### 2.2 Microscopic agglutination test (MAT)

All serum samples were subjected to MAT in duplicate to determine the exposure of individual animals to the various serovars of *L. interrogans* bacteria (Niloofa et al., 2015; Sakhaee, 2011; Sakhaee et al., 2010). A seven to 10 days’ culture of different serovars of *L. interrogans* in a liquid medium (GRA‐Sina, Sinajen, Tehran, Iran) was used as a source of cellular antigens. The density of leptospires was checked using a counting chamber (Petroff‐Hauser USA) and adjusted to 2 × 10^8^ cell/ml. All serum samples were serially diluted in phosphate buffer solution (PBS), starting from 1 in 50 dilutions, using twofold dilution (1 in 100, 200, 400, 800 and 1600). Then, 10 μl of serum dilution was added to 10 μl of the appropriate antigen in a 96‐well plate and incubated at 30°C for 90 min. Upon completion of incubation, samples from individual wells were transferred to a microscopic slide and examined using a dark‐field microscope (Olympus B×50). One antigen control and two (positive and negative) standard serum controls were used for each 96 well plate (Sakhaee, 2011). Samples with 50% agglutination were considered positive. According to the OIE Terrestrial Manual, a titre of 1:100 diluted serum is interpreted as positive and serves international trade purposes (World Organization of Animal Health‐OIE, 2019). All samples were tested against nine leptospiral antigens (*L. interrogans serovar Hardjo*, *L. interrogans serovar Tarasovi*, *L. interrogans serovar Australis*, *L. interrogans serovar Icterohaemorrhagiae*, *L. interrogans serovar Pomona*, *L. interrogans serovar Grippotyphosa*, *L. interrogans serovar Autumnalis*, *L. interrogans serovar Canicola* and *L. interrogans serovar Ballum*).

### 2.3 Statistical analysis

Fisher exact testing was conducted for comparing the leptospiral seroprevalence among the different animals using SPSS software (version 20).

## 3 RESULTS

Among the 77 samples studied, 44.1% reacted positively to at least one serovar‐specific antigen preparation of *L. interrogans*. The rate of positive reactions was higher in stray dogs versus wild canines (55.2% and 37.5%), but this was not statistically significant (*p = *0.159). The highest detected frequency was for *L. interrogans serovar Grippotyphosa* (*n* = 14) and *L. interrogans serovar Canicola* (*n* = 11) (Table 1).

**TABLE 1:** Distribution of positive MAT results as measured in canine serum samples

Type of canines	Positive/total cases (%)	Number	Serovar						
			Hardjo	Australis	Tarasovi	Canicola	Ictero.	Pomona	Grippo.
			titre						
Stray dogs	18/48 (37.5)	7							100
		1					100		
		6^**^				100–1600			
		2				100			100
		1	100			100			200
		1	100						200
Jackal/fox	16/29 (55.2)	1^*^		400			800	800	
		1^*^		100		100			
		1^*^				100			
		1^*^			200				400
		3						100	
		2							100
		2			100				
		2					100		
		1		200					
		2		100				100	
Total	48/77 (44.1)		2	5	3	11	4	6	14

*Jackal.

**Titre 200 = 3 cases, Titre 100 = 1 case and Titre 1600 = 2 cases.

Hardjo: *L. interrogans serovar Hardjo*, Australis: *L. interrogans serovar Australis*, Tarasovi: *L. interrogans serovar Tarasovi*, Canicola: *L. interrogans serovar Canicola*, Ictero: *L. interrogans serovar Icterohaemorrhagiae*, Pomona: *L. interrogans serovar Pomona*, Grippo: *L. interrogans serovar Grippotyphosa*.

Positive reactions with *L. interrogans serovar Pomona* (*n* = 6), *L. interrogans serovar Australis* (*n* = 5) and *L. interrogans serovar Tarasovi* (*n* = 3) were detected only among sera collected from jackals and foxes. No positive reaction against *L. interrogans serovar Canicola* was detected among foxes. Among the stray dogs, the highest frequency of positivity was for *L. interrogans serovar Grippotyphosa* (*n* = 11/18, 61.1%) and *L. interrogans serovar Canicola* (*n* = 9/18, 50%). Antibodies against *L. interrogans serovar Hardjo* (*n* = 2) were detected among stray dogs only (Table 1).

The highest detected titre was 1:1600 for *L. interrogans serovar Canicola* in two stray dogs and a titre of 1:800 against *L. interrogans serovar Icterohaemorrhagiae* and *L. interrogans serovar Pomona* in a single jackal (Table 1). Among dog samples, a positive triple reaction against *L. interrogans serovar Hardjo* (1:100), *L. interrogans serovar Canicola* (1:100) and *L. interrogans serovar Grippotyphosa* (1:200) was spotted. All tested jackal samples showed positive reactions. A single jackal sample revealed a response at 1:800 against *L. interrogans serovar Icterohaemorrhagiae* and *L. interrogans serovar Pomona* followed by 1:400 against *L. interrogans serovar Australis* (Table 1).

## 4 DISCUSSION

Leptospirosis is one of the most significant re‐emerging infectious diseases in Iran (Parhizgari et al., 2017). The present study investigated the prevalence of antibodies against serovar‐specific leptospiral antigens among wild canines and stray dogs using the MAT (Niloofa et al., 2015). Clinical samples were collected from animals captured in the North Khorasan province in Iran. The location of the study is in a mountainous area with 9 months of cold weather. Conditions such as these should always be considered in our type of study but the precise impact of climate is ill defined.

We report specific antibodies against various *L. interrogans* serovars found in jackals and foxes. The prevalence of specific antibodies against those leptospiral serovars detected among foxes in this study (55.2%) is higher than the prevalence reported in native foxes in Chile (7.7%) (Galarce et al., 2021), red foxes from Spain (47.1%) (Millán et al., 2009) and Croatia (33.8% and 31.25%) (Slavica et al., 2008; Slavica et al., 2011). The prevalence was slightly lower than recorded among red foxes from Croatia (57.6%) (Milas et al., 2006). In a recently conducted study on jackals, Nakonechnyi et al. (2019) found positive reactions against *Leptospira interrogans* among all nine animals that were tested.

The documented overall prevalence of 37.5% among dogs in this study is almost 2.5‐fold higher than the estimated overall prevalence of 14.6% (95% CI: 3.49–25.77) for dogs in Iran (Khalili et al., 2020). The highest recorded prevalence was from Tehran (32.6%) (Rad et al., 2004), following Alborz (21.8%) (Fahimipour et al., 2021), Khorasan Razavi (14.3%) (Kamrani & Sardari, 2003), West Azerbaijan (6.4%) (Hayatrohi et al., 2014) and Khuzestan (5.4%) (Avizeh et al., 2009). The serological prevalence reported by the current study is in the mean but broad range among those reported from other regions of the world (8% to 74.2%) (Table 2). The high prevalence of leptospiral antibodies among stray dogs conceivably results from the greater risk for leptospirosis rising from spending all of their time outdoors and in urban environments as reported for large working and hunting dogs in the United States (Adin & Cowgill, 2000; Alton et al., 2009; Birnbaum et al., 1998; Ward et al., 2002).

**TABLE 2:** Prevalence and the most dominant leptospiral serovars reported from Iran and other region of world among dogs

	Study place	Reported prevalence %	Serovars	
Iranian province	North Khorasan	37.5	*L. interrogans Grippotyphosa* (61.1%) and *L. interrogans Canicola* (50%)	Current study
	Alborz	21.8	*L. interrogans Canicola* (33.3%), *L. interrogans Icterohaemorrhagiae* (25%) and *L. interrogans Grippotyphosa* (20.83%)	Fahimipour et al. (2021)
	Khuzestan	5.4	*L. interrogans Hardjo* (44.5%), *L. interrogans Ballum* and *L. interrogans Icterohaemorrhagiae* (22.2%)	Avizeh et al. (2009)
	Khorasan Razavi	14.3	*L. interrogans Canicola* (11.98%), *L. interrogans Pomona* (4.79%) and *L. interrogans Hardjo* (2.39%)	Kamrani and Sardari (2003)
	West Azerbaijan	6.4	*L. interrogans Hardjo* (44.5%), *L. interrogans Icterohaemorrhagiae* and *Ballum* (22.2%),	Hayatrohi et al. (2014)
	Tehran	32.6	*L. interrogans Canicola* (9%), *L. interrogans Icterohaemorrhagiae* (5.7%) and *L. interrogans Grippotyphosa* (3.7%)	Rad et al. (2004)
Other countries	Thailand	12.1	*L. interrogans Sejroe* (4.4%) and *L. interrogans Icterohaemorrhagiae* (3.7%),	Altheimer et al. (2020)
	India	71.12	*L. interrogans Autumnalis* (23.9%) and *L. interrogans Australis* (19.17%)	Ambily et al. (2013)
	Turkey	43.96	*L. interrogans Bratislava* (66%) and *L. interrogans Canicola* (21.5%)	Aslantaş et al. (2005)
	Sudan	74.2	*L. interrogans Autumnalis* (>70%) and *L. interrogans Icterohaemorrhagiae* (> 60%%)	Roqueplo et al. (2015)
	Gabon	34.6	*L. interrogans Autumnalis* (>30%) and *L. interrogans Icterohaemorrhagiae* (> 70%)	Roqueplo et al. (2015)
	Ivory Coast	58.1	*L. interrogans Autumnalis* (60%) and *L. interrogans Grippotyphosa* (40%)	Roqueplo et al. (2015)
	Egypt	11.3	*L. interrogans Icterohaemorrhagiae* (47.3%) and *L. interrogans Canicola* (52.6%)	Samir et al. (2015)
	Germany	32	*L. interrogans Australis* (24%), *L. interrogans Grippotyphosa* (20%) and *L. interrogans Pomona* (9%).	Mayer‐Scholl et al. (2013)
	Switzerland	28.1	*L. interrogans Australis* (70.5%) and *L. interrogans Bratislava* (69.1%)	Major et al. (2014)
	Spain	25.8	*L. interrogans Icterohaemorrhagiae* (19.4%) and *L. interrogans Bratislava* (8.5%)	López et al. (2019)
	Italy	29.9	*L. interrogans Icterohaemorrhagiae* (57%) and *L. interrogans Bratislava* (22%)	Piredda et al. (2021)
	Italy	49	*L interrogans Australis* (39.3%) and *L. interrogans Icterohaemorrhagiae* (32.1%)	Tagliabue et al. (2016)
	Canada	8	*L. interrogans Autumnalis* (31.3%) and *L. interrogans Bratislava* (15.8%)	Alton et al. (2009)
	Chile	25.1	*L. interrogans Canicola* (51.6 %)	Lelu et al. (2015)

The causative agents of leptospirosis among dogs are usually the serovars *L. interrogans serovar Canicola*, *L. interrogans serovar Icterohaemorrhagiae*, *L. interrogans serovar Grippotyphosa*, *L. interrogans serovar Pomona* and *L. interrogans serovar Bratislava* (Klaasen & Adler, 2015). The high detection frequency for *L. interrogans serovar Grippotyphosa* (61.1%) and *L. interrogans serovar Canicola* (50%) and the lack of positivity against *L. interrogans serovar Pomona* among dogs in the current study is similar to the results of a study conducted by Fahimipour et al. (2019) in the Alborz province. These authors reported a high prevalence of *L. interrogans serovar Canicola* (33.3%), *L. interrogans serovar Icterohaemorrhagiae* (25%) and *L. interrogans serovar Grippotyphosa* (20.83%), followed by a low prevalence of *L. interrogans serovar Pomona* (4.1%) (Fahimipour et al., 2021). Studies conducted in other regions of Iran including Tehran (Rad et al., 2004), Khuzestan (Avizeh et al., 2009), West Azerbaijan (Hayatrohi et al., 2014) and a study in a nearby region of the current study in Khorasan Razavi (Kamrani & Sardari, 2003) illustrated positive reactions of sera with different serovars of *L. interrogans* (Table 2) (Figure 1). The common serovars among dogs reported here differ from those found in Asian, African, European and American countries (Table 2).

Based on serological data collected during the current study, we conclude that dogs, foxes and jackals are natural reservoirs of Leptospira in the area that we covered. Specific antibodies against *L. interrogans* were reported as was done among rodents in the same geographical regions before (Arzamani et al., 2018). Dogs are considered an important host for *Leptospira interrogans*, and having close contact with stray dogs may increase the risk of infection in humans (Lelu et al., 2015).

## 5 CONCLUSION

We demonstrate that leptospirosis is a significant endemic epizootic disease in the study region. More extensive investigations on the large population of wild canines is recommended for better understanding the possible transfer of infections from wild and domesticated animals to humans. First thing to better substantiate this risk would be to perform seroprevalence studies among humans from which in the end prophylactic measures may be designed.

## CONFLICT OF INTEREST

The authors have no relevant financial or non‐financial interests to disclose.

## FUNDING INFORMATION

The authors declare that no funds, grants or other support were received during the preparation of this manuscript.

## ETHICS STATEMENT

Sample collection was performed according to the rules and regulations set by the Ethical Committee of North Khorasan University of Medical Sciences (project number: IR. NKUMS. REC1391.017.).

## AUTHOR CONTRIBUTIONS

Kourosh Arzamani: Conceptualization, Methodology, Resources

Gholamreza Abdollahpour and Amir Azimian: Investigation

Hamed Ghasemzadeh‐Moghaddam: Visualization, Writing – Original Draft Preparation

Alex van Belkum: Writing – Review & Editing

### PEER REVIEW

The peer review history for this article is available at https://publons.com/publon/10.1002/vms3.890.

## Data Availability

Data openly available in a public repository that issues datasets with DOIs.

## References

[vms3890-bib-0001] Ab Rahman, M. H. A. , Hairon, S. M. , Hamat, R. A. , Jamaluddin, T. Z. M. T. , Shafei, M. N. , Idris, N. , Osman, M. , Sukeri, S. , Wahab, Z. A. , Mohammad, W. M. Z. W. , Idris, Z. , & Daud, A. (2018). Seroprevalence and distribution of leptospirosis serovars among wet market workers in northeastern Malaysia: A cross sectional study. BMC Infectious Diseases, 18(1), 569–273. 30428852 10.1186/s12879-018-3470-5 PMC6236877

[vms3890-bib-0002] Adler, B. , & de la Peña Moctezuma, A. (2010). Leptospira and leptospirosis. Veterinary Microbiology, 140(3‐4), 287–296. 19345023 10.1016/j.vetmic.2009.03.012

[vms3890-bib-0003] Adin, C. A. , & Cowgill, L. D. (2000). Treatment and outcome of dogs with leptospirosis: 36 cases (1990–1998). Journal of the American Veterinary Medical Association, 216(3), 371–375. 10668536 10.2460/javma.2000.216.371

[vms3890-bib-0004] Altheimer, K. , Jongwattanapisan, P. , Luengyosluechakul, S. , Pusoonthornthum, R. , Prapasarakul, N. , Kurilung, A. , Broens, E. M. , Wagenaar, J. A. , Goris, M. G. A. , Ahmed, A. A. , Pantchev, N. , Reese, S. , & Hartmann, K. (2020). Leptospira infection and shedding in dogs in Thailand. BMC Veterinary Research, 16(1), 1–13. 32178664 10.1186/s12917-020-2230-0 PMC7077098

[vms3890-bib-0005] Alton, G. D. , Berke, O. , Reid‐Smith, R. , Ojkic, D. , & Prescott, J. F. (2009). Increase in seroprevalence of canine leptospirosis and its risk factors, Ontario 1998–2006. Canadian Journal of Veterinary Research, 73(3), 167–175. 19794888 PMC2705070

[vms3890-bib-0006] Ambily, R. , Mini, M. , Joseph, S. , Krishna, S. V. , & Abhinay, G. (2013). Canine leptospirosis‐a seroprevalence study from Kerala, India. Veterinary World, 6(1), 41–42.

[vms3890-bib-0007] Arzamani, K. , Mohammadi, Z. , Shirzadi, M. R. , Alavinia, S. M. , Jafari, B. , & Darvish, J. (2018). Faunistic study of the rodents of north Khorasan province, north east of Iran, 2011–2013. Journal of Arthropod‐Borne Diseases, 12(2), 127–134. 30123806 PMC6091794

[vms3890-bib-0008] Aslantaş, Ö. , Özdemir, V. , Kiliç, S. , & Babür, C. (2005). Seroepidemiology of leptospirosis, toxoplasmosis, and leishmaniosis among dogs in Ankara, Turkey. Veterinary Parasitology, 129(3‐4), 187–191. 15845273 10.1016/j.vetpar.2004.11.037

[vms3890-bib-0009] Avizeh, R. , Ghorbanpoor, M. , Hatami, S. , & Abdollahpor, G. (2009). Seroepidemiology of canine leptospirosis in Ahvaz, Iran. Iranian Journal of Veterinary Medicine, 2(2), 75–79.

[vms3890-bib-0010] Azocar‐Aedo, L. , Monti, G. , & Jara, R. (2017). Similar prevalence of anti‐Leptospira antibodies in domestic dogs from urban and rural areas in southern Chile: A public health concern. Journal of Preventive Medicine and Public Health, 1, 1–5.

[vms3890-bib-0011] Birnbaum, N. , Barr, S. , Center, S. , Schermerhorn, T. , Randolph, J. , & Simpson, K. (1998). Naturally acquired leptospirosis in 36 dogs: Serological and clinicopathological features. Journal of Small Animal Practice, 39(5), 231–236. 9631358 10.1111/j.1748-5827.1998.tb03640.x

[vms3890-bib-0012] Fahimipour, A. , Khaki, P. , & Moradi Bidhendi, S. (2019). Seroepidemiology of leptospira infection in stray dogs by Mat during a one‐year period from rural communities of Koohsar, Alborz, Iran. In Proceedings of the 19th International Congress of Microbiology of Iran, Tehran . https://civilica.com/doc/783022

[vms3890-bib-0013] Fahimipour, A. , Khaki, P. , & Moradi Bidhendi, S. (2021). Seroepidemiological analysis of leptospiral infection using MAT in stray dogs in Alborz, Iran. Archives of Razi Institute, 76(2), 391–396. 34223737 10.22092/ari.2020.127253.1376 PMC8410190

[vms3890-bib-0014] Fratini, F. , Bertelloni, F. , & Cilia, G. (2020). Leptospira infection in wild animals. Hauppauge, NY, USA: Nova Science Publisher. ISBN 978‐1‐53618‐222‐4.

[vms3890-bib-0015] Galarce, N. , de la Fuente, S. , Escobar, B. , Dettleff, P. , Abalos, P. , Hormazábal, J. C. , Flores, R. , Sallaberry‐Pincheira, N. , & Martínez, V. (2021). Survey of zoonotic bacterial pathogens in native foxes in central Chile: First record of *Brucella canis* exposure. Animals, 11(7), 1980. 34359107 10.3390/ani11071980 PMC8300164

[vms3890-bib-0016] Hashemi, S.‐A. , Arzamani, K. , Abdollahpour, G. , Beheshti, N. , Alavinia, M. , Azimian, A. , Neela, V. K. , van Belkum, A. , & Ghasemzadeh‐Moghaddam, H. (2021). Seroprevalence of Leptospira infection in occupational risk groups in North Khorasan province, Iran. Heliyon, 7(1), e05983. 33506135 10.1016/j.heliyon.2021.e05983 PMC7814158

[vms3890-bib-0017] Hayatrohi, A. , Lak, A. G. , Hashempour, A. , Gholizadeh, S. S. , Abdollahpour, G. , & Zadeh, R. (2014). Survey on S eroprevalence of Leptospira serotypes in household dogs using MAT method in Urmia, Iran. Bulletin of Environment, Pharmacology, and Life Sciences, 3, 158–162.

[vms3890-bib-0018] Jamshidi, S. , Vandyousefi, J. , Dezfoulian, O. , & Selk Ghaffari, M. (2008). Isolation of *Leptospira canicola* from a dog in Iran: First report. Iranian Journal of Veterinary Research, 9(3), 291–294.

[vms3890-bib-0019] Kamrani, A. , & Sardari, K. (2003). Seroprevalence of leptospiral antibodies in stray dogs in Mashhad, Iran. In Proceedings of the World Small Animal Veterinary Association World Congress . Bangkok, Thailand.

[vms3890-bib-0020] Khalili, M. , Sakhaee, E. , Amiri, F. B. , Safat, A. A. , Afshar, D. , & Esmaeili, S. (2020). Serological evidence of leptospirosis in Iran: A systematic review and meta‐analysis. Microbial Pathogenesis, 138, e103833. 10.1016/j.micpath.2019.103833 31698052

[vms3890-bib-0021] Klaasen, H. L. E. , & Adler, B. (2015). Recent advances in canine leptospirosis: Focus on vaccine development. Veterinary Medicine: Research and Reports, 6, 245–260. 10.2147/VMRR.S59521 PMC6067773 30101111

[vms3890-bib-0022] Lelu, M. , Muñoz‐Zanzi, C. , Higgins, B. , & Galloway, R. (2015). Seroepidemiology of leptospirosis in dogs from rural and slum communities of Los Rios Region, Chile. BMC Veterinary Research, 11(1), 1–9. 25880871 10.1186/s12917-015-0341-9 PMC4329218

[vms3890-bib-0023] López, M. C. , Vila, A. , Rodón, J. , & Roura, X. (2019). Leptospira seroprevalence in owned dogs from Spain. Heliyon, 5(8), e02373. 31485543 10.1016/j.heliyon.2019.e02373 PMC6717157

[vms3890-bib-0024] Major, A. , Schweighauser, A. , & Francey, T. (2014). Increasing incidence of canine leptospirosis in Switzerland. International Journal of Environmental Research and Public Health, 11(7), 7242–7260. 25032740 10.3390/ijerph110707242 PMC4113873

[vms3890-bib-0025] Mayer‐Scholl, A. , Luge, E. , Draeger, A. , Nöckler, K. , & Kohn, B. (2013). Distribution of Leptospira serogroups in dogs from Berlin, Germany. Vector‐Borne and Zoonotic Diseases, 13(3), 200–202. 23428087 10.1089/vbz.2012.1121

[vms3890-bib-0026] Milas, Z. , Turk, N. , Janicki, Z. , Slavica, A. , Starešina, V. , Barbić, L. , Lojkić, M. , & Modrić, Z. (2006). Leptospiral antibodies in red foxes (Vulpes vulpes) in northwest Croatia. Veterinarski Arhiv, 76, 51–57.

[vms3890-bib-0027] Millán, J. , Candela, M. G. , López‐Bao, J. V. , Pereira, M. , Jiménez, M. Á. , & León‐Vizcaíno, L. (2009). Leptospirosis in wild and domestic carnivores in natural areas in Andalusia, Spain. Vector‐Borne and Zoonotic Diseases, 9(5), 549–554. 18973450 10.1089/vbz.2008.0081

[vms3890-bib-0028] Nakonechnyi, I. , Perots'ka, L. , Pyvovarova, I. , & Chornyi, V. (2019). Ecological and epizootic roles of Golden jackal, genus *Canis aureus* in the Northwest of Black Sea coast. Scientific Messenger of LNU of Veterinary Medicine and Biotechnologies. Series: Veterinary Sciences, 21(94), 37–43.

[vms3890-bib-0029] Niloofa, R. , Fernando, N. , de Silva, N. L. , Karunanayake, L. , Wickramasinghe, H. , Dikmadugoda, N. , Premawansa, G. , Wickramasinghe, R. , de Silva, H. J. , Premawansa, S. , Rajapakse, S. , & Handunnetti, S. (2015). Diagnosis of leptospirosis: Comparison between microscopic agglutination test, IgM‐ELISA and IgM rapid immunochromatography test. PLoS One, 10(6), e0129236. 26086800 10.1371/journal.pone.0129236 PMC4472754

[vms3890-bib-0030] Parhizgari, N. , Gouya, M. M. , & Mostafavi, E. (2017). Emerging and re‐emerging infectious diseases in Iran. Iranian Journal of Microbiology, 9(3), 122–142. 29225752 PMC5719507

[vms3890-bib-0031] Piredda, I. , Ponti, M. N. , Piras, A. , Palmas, B. , Pintore, P. , Pedditzi, A. , & Chisu, V. (2021). New insights on leptospira infections in a canine population from North Sardinia, Italy: A sero‐epidemiological study. Biology, 10(6), 507. 34200298 10.3390/biology10060507 PMC8226461

[vms3890-bib-0032] Rad, M. , Zeynali, A. , Tabatabayi, A. , Bokaei, S. , & Vandi, Y. J. (2004). Seroprevalence and bacteriological study of canine leptospirosis in Tehran and its suburban areas. Iranian Journal of Veterinary Research, 5(2), 53–80.

[vms3890-bib-0033] Roqueplo, C. , Marié, J.‐L. , André‐Fontaine, G. , Kodjo, A. , & Davoust, B. (2015). Serological survey of canine leptospirosis in three countries of tropical Africa: Sudan, Gabon and Ivory Coast. Comparative Immunology, Microbiology and Infectious Diseases, 38, 57–61. 25467033 10.1016/j.cimid.2014.10.006

[vms3890-bib-0034] Sakhaee, E. (2011). Detection of Leptospiral antibodies by microscopic agglutination test in north–east of Iran. Asian Pacific Journal of Tropical Biomedicine, 1(3), 227–229. 23569764 10.1016/S2221-1691(11)60032-4 PMC3609195

[vms3890-bib-0035] Sakhaee, E. , Abdollahpour, G. , Bolourchi, M. , & Tabrizi, S. S. (2010). Comparison between microscopic agglutination test (MAT) and enzyme‐linked immunosorbent assay (ELISA) for detection of leptospiral antibodies in cattle. Comparative Clinical Pathology, 19(1), 5–9.

[vms3890-bib-0036] Samir, A. , Soliman, R. , El‐Hariri, M. , Abdel‐Moein, K. , & Hatem, M. E. (2015). Leptospirosis in animals and human contacts in Egypt: Broad range surveillance. Revista da Sociedade Brasileira de Medicina Tropical, 48, 272–277. 26108004 10.1590/0037-8682-0102-2015

[vms3890-bib-0037] Shi, D. , Liu, M. , Guo, S. , Liao, S. , Sun, M. , Liu, J. , Wang, L. , Wang, Z. , Wang, S. , Yang, D. , & Chai, T. (2012). Serological survey of canine leptospirosis in southern China. Pakistan Veterinary Journal, 32(2), 280–282.

[vms3890-bib-0038] Slavica, A. , Cvetnić, Ž. , Milas, Z. , Janicki, Z. , Turk, N. , Konjević, D. , Toncic, J. , & Lipej, Z. (2008). Incidence of leptospiral antibodies in different game species over a 10‐year period (1996–2005) in Croatia. European Journal of Wildlife Research, 54(2), 305–311.

[vms3890-bib-0039] Slavica, A. , Dezdek, D. , Konjevic, D. , Cvetnic, Z. , Sindicic, M. , Stanin, D. , Habus, J. , & Turk, N. (2011). Prevalence of leptospiral antibodies in the red fox (*Vulpes vulpes*) population of Croatia. Veterinarni Medicina, 56(4), 209–213.

[vms3890-bib-0040] Tagliabue, S. , Figarolli, B. M. , D'Incau, M. , Foschi, G. , Gennero, M. S. , Giordani, R. , Giordani, R. , Natale, A. , Papa, P. , Ponti, N. , Scaltrito, D. , Spadari, L. , Vesco, G. , & Ruocco, L. (2016). Serological surveillance of Leptospirosis in Italy: Two‐year national data (2010–2011). Veterinaria italiana, 52, 129–138. 27393874 10.12834/VetIt.58.169.2

[vms3890-bib-0041] Torkan, S. , & Momtaz, H. (2019). Molecular detection of leptospira species serotypes in Iranian stray dogs. International Journal of Medical Laboratory, 6(2), 138–142.

[vms3890-bib-0042] Ullmann, L. , & Langoni, H. (2011). Interactions between environment, wild animals and human leptospirosis. Journal of Venomous Animals and Toxins including Tropical Diseases, 17(2), 119–129.

[vms3890-bib-0043] Ward, M. P. , Glickman, L. T. , & Guptill, L. F. (2002). Prevalence of and risk factors for leptospirosis among dogs in the United States and Canada: 677 cases (1970–1998). Journal of the American Veterinary Medical Association, 220(1), 53–58. 12680448 10.2460/javma.2002.220.53

[vms3890-bib-0044] World Organization of Animal Health (OIE) (2019). Leptospirosis. In Manual of diagnostic tests and vaccines for terrestrial animals (pp. 503–516). OIE.

